# Task set and instructions influence the weight of figural priors: A psychophysical study with extremal edges and familiar configuration

**DOI:** 10.3758/s13414-021-02282-5

**Published:** 2021-04-20

**Authors:** Tandra Ghose, Mary A. Peterson

**Affiliations:** 1 Department of Psychology, University of Kaiserslautern, 67663 Kaiserslautern, Germany; 2 Department of Psychology; Cognitive Science Program, University of Arizona, Tucson, AZ USA

**Keywords:** Perceptual organization, Figure–ground perception, Extremal edges, Familiar configuration, Task instructions, Task set, Shape perception, Figural priors, Depth ordering, Familiar parts

## Abstract

In figure–ground organization, the figure is defined as a region that is both “shaped” and “nearer.” Here we test whether changes in task set and instructions can alter the outcome of the cross-border competition between figural priors that underlies figure assignment. Extremal edge (EE), a relative distance prior, has been established as a strong figural prior when the task is to report “which side is nearer?” In three experiments using bipartite stimuli, EEs competed and cooperated with familiar configuration, a shape prior for figure assignment in a “which side is shaped?” task.” Experiment 1 showed small but significant effects of familiar configuration for displays sketching upright familiar objects, although “shaped-side” responses were predominantly determined by EEs. In Experiment 2, instructions regarding the possibility of perceiving familiar shapes were added. Now, although EE remained the dominant prior, the figure was perceived on the familiar-configuration side of the border on a significantly larger percentage of trials across all display types. In Experiment 3, both task set (nearer/shaped) and the presence versus absence of instructions emphasizing that familiar objects might be present were manipulated within subjects. With familiarity thus “primed,” effects of task set emerged when EE and familiar configuration favored opposite sides as figure. Thus, changing instructions can modulate the weighing of figural priors for shape versus distance in figure assignment in a manner that interacts with task set. Moreover, we show that the influence of familiar parts emerges in participants without medial temporal lobe/ perirhinal cortex brain damage when instructions emphasize that familiar objects might be present.

## Introduction

### Figure assignment: Near and shaped

Perceptual organization is the process by which bits and pieces of visual information are organized into meaningful units. Figure–ground perception is one outcome of perceptual organization, in which a border shared by two regions is assigned to one side only. The side to which the border is assigned is perceived as a figure that is both *shaped* and *nearer* to the observer than the opposite side, which is locally shapeless and seems to continue behind the figure (e.g., Hochberg, 1971; Palmer, 1999). The importance of this topic lies in the fact that 2D projections of overlapping objects in the 3D world share a border. Therefore, border assignment processes are ubiquitous.

**Fig. 1:**
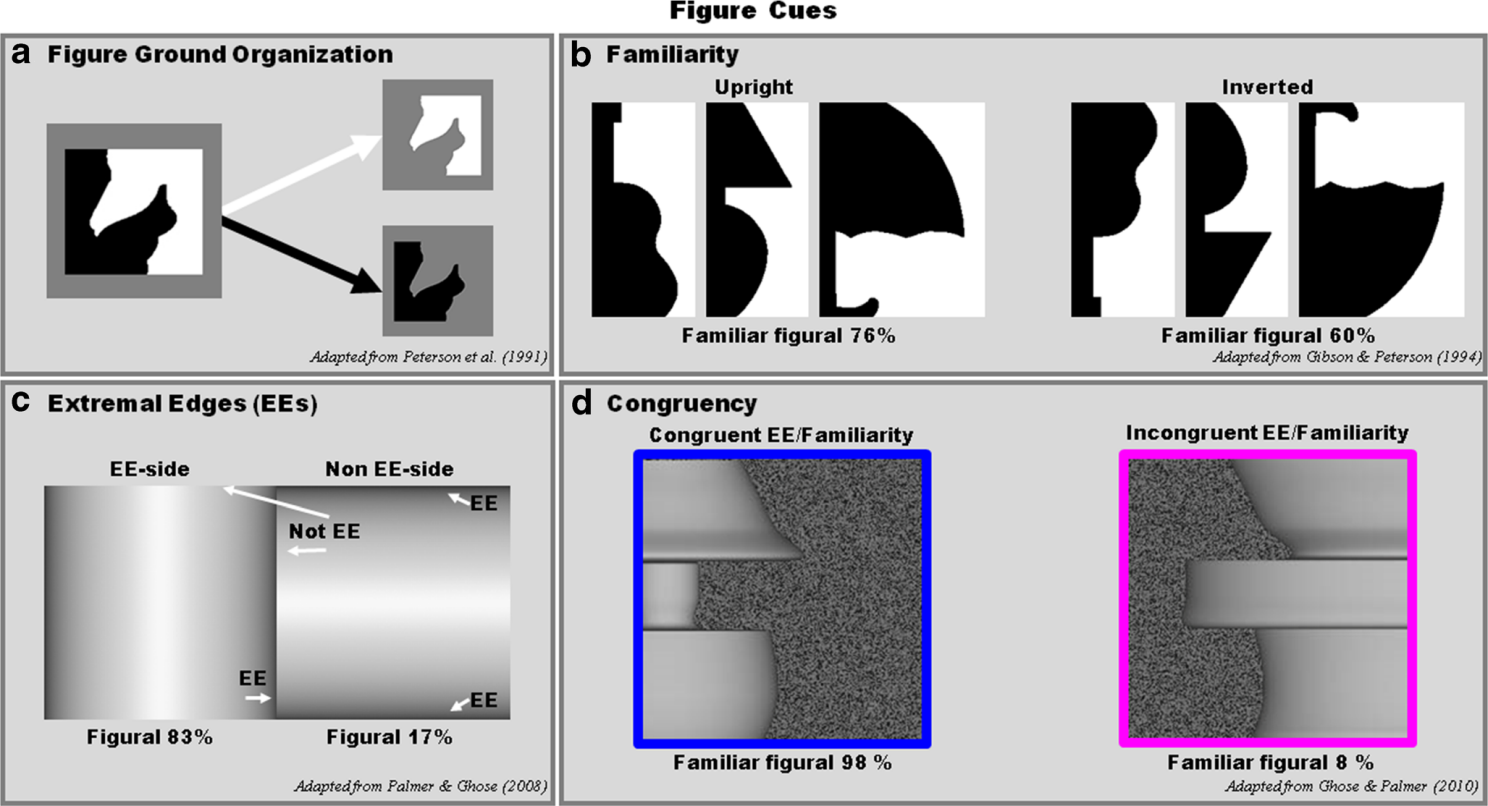
Figural priors. **a** Figure–ground organization: The “figure” is the side of a bipartite image that appears to be “shaped by the shared contour, nearer to the observer, and thing-like.” In the image on the left, black and white equal area regions share a border. The top and bottom insets illustrate the shaped figures that would be perceived if the border were assigned to the white side (top) or the black side (bottom). The region on the opposite side of the border would simply appear to continue behind the shaped figure. **b** Familiar configuration. Intact upright familiar configuration is a prior for figural assignment (%figural = 76%), while inverted ones are not as strong (%figural = 60%; adapted from Gibson & Peterson, 1994). **c** Extremal edges (EE). EEs arise when a 3D convex surface curves around to occlude a part of itself. For the EE side (on left), the steepest part of the shading gradient is parallel to the shared border, thus specifying that the left side is closer than the right side. The side for which the shared border is an extremal edge is seen as figural 83% of the time, even when 3D convexity is the same for the two sides sharing the border, as in **c** (adapted from Palmer & Ghose, 2008). **d** Congruent versus incongruent. EE alters the probability that the figure is perceived on the familiar configuration side of a border depending on whether it is cooperating (98%) or competing (8%) with familiarity (adapted from Ghose & Palmer, 2010) in a “Which side is closer?” task

Figural priors, also known as “figural cues,” increase the likelihood that one side of a shared border will be perceived as the figure. Some of these priors are image-based shape priors, such as smaller size, edge convexity, symmetry, surroundedness, and top-bottom polarity (Bahnsen, 1928; Hulleman & Humphreys, 2004; Kanizsa & Gerbino, 1976; Metzger, 2006; Rubin, 1915; Rubin, 1958). An experience-based shape prior is familiar configuration: Figures are likely to be perceived on the side of a border where a portion of a well-known object (i.e., a familiar configuration) is sketched (e.g., Navon, 2011; Peterson et al., 1991; Peterson & Gibson, 1994a, 1994b; Vecera & Farah, 1997). Other figural priors are relative distance priors (cf. Palmer, 1999; e.g., extremal edges, folds, and gradient cuts; Ghose & Palmer, 2010, 2016; Kim & Anstis, 2016; Kunsberg et al., 2018; Palmer & Ghose, 2008), edge-region grouping (Palmer & Brooks, 2008), and the depth cue of binocular disparity (e.g., Burge et al., 2005; Grossberg, 2016; Qiu & von der Heydt, 2005). Figure 1a–c illustrates figure–ground organization and the figural priors of familiar configuration and extremal edges.

In what follows, we summarize the current evidence regarding the two figural priors investigated in this study—familiar configuration and extremal edges (EEs). This section is followed by a brief review of the evidence suggesting that figure–ground organization entails competition between figural priors present on opposite sides of a shared border. In the experiments reported here, we examine what is perceived when EE and familiar configuration cooperate and compete, paying particular attention to whether task set alters the outcome. Accordingly, a brief discussion of the role of task set in perception also precedes the presentation of the experiments.

### 2D familiar configuration

Gestalt psychologists (e.g., Koffka, Köhler, and Wertheimer) and traditional information-processing theories of visual perception held that only those regions in the visual input that had been determined to be figures are processed for meaning and matched to memory representations; regions determined to be grounds are not (Palmer & Rock, 1994a, 1994b; for an opposing view, which was not much pursued, see Rubin, 1915; Rubin, 1958; Sander, 1930; for a review, see Peterson, Cacciamani, Mojica, & Sanguinetti, 2012). However, it has been shown that the side of a border that depicts a well-known (or “familiar”) meaningful object based on past experience is more likely to be perceived as figural than the complementary side (Peterson, 1994; Peterson et al., 1991; Peterson & Gibson, 1991, 1993, 1994a, 1994b). In the experiments demonstrating these effects, the familiar objects were depicted in the orientation in which they are typically experienced (their canonical upright orientation). Therefore, Peterson et al. hypothesized that familiarity would be shown to influence figure assignment if observers were more likely to perceive the figure on the side of the central border where a familiar configuration was depicted when it was present in its upright orientation rather than an inverted orientation; this orientation manipulation held constant for all other known figural priors (see Fig. 1b). Indeed, when the displays were rotated by 180 degrees, the effect of familiarity was diminished (Gibson & Peterson, 1994; Peterson & Gibson, 1991, 1993, 1994a, 1994b; Peterson et al., 1991; for a review, see Peterson, 1994, 2019). The orientation dependency of these familiarity effects indicated that the relevant object memories must be activated quickly in order to influence figure–ground assignment, as object memories are activated by inverted versions of familiar objects, albeit later in time (Jolicoeur, 1985, 1988; Perrett et al., 1998; Tarr & Pinker, 1989).

**Fig. 2:**
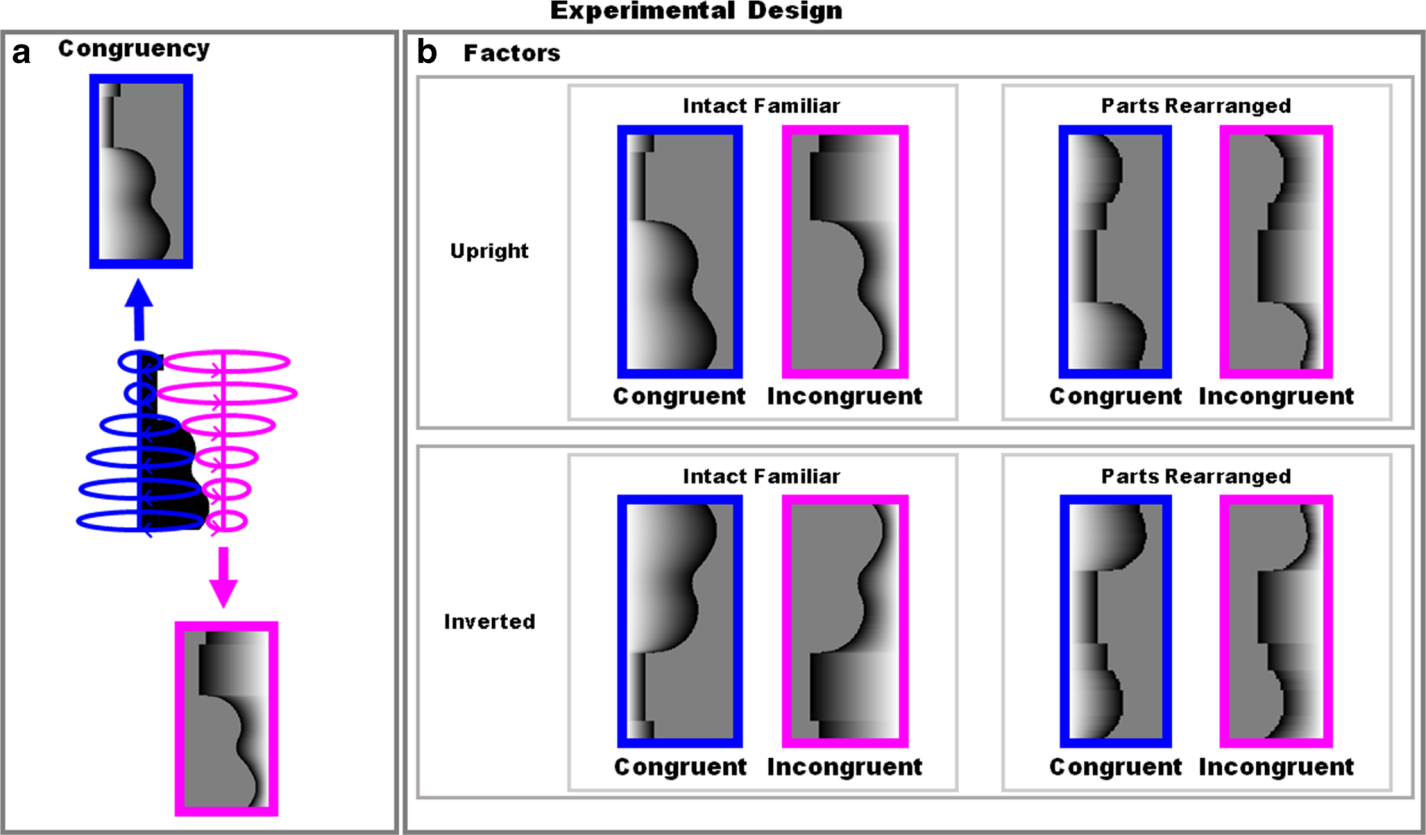
Experimental design. **a** Congruency of familiarity and EE: Surfaces of revolution were created by rotating the shared contour around different axes to create congruent (blue) and incongruent (pink) versions from a given black-and-white image. **b** Factors: Orientation upright versus inverted. Configuration type: Intact familiar where the shared border elicits good match to a memory representation of a well-known object. Part rearranged: Novel configuration created by spatially rearranging parts of familiar configuration (in the example shown, the neck of the guitar separates the parts above and below its waist). Congruency: Congruent—EE and familiarity present on the same side of the shared border (blue frames). Incongruent—EE and familiarity are present on the opposite sides of the shared border (pink frames)

Peterson and colleagues showed that familiar parts alone were insufficient for these effects by comparing performance with displays depicting intact versions of upright familiar configurations on one side of a border (the “critical” side) to performance with matched displays in which the critical side of the border depicted novel configurations created by spatially rearranging the same parts (e.g., for the guitar profile in Fig. 1b, the part-rearranged version depicted the neck of the guitar separating the parts above and below its waist; see Fig. 2b). They found that the figure was substantially and significantly more likely to be perceived on the critical side of the borders where intact familiar configurations were depicted rather than novel configurations created by rearranging the parts (Peterson et al., 1991; Peterson et al., 1998; Peterson et al., 2000). Thus, intact familiar configurations in their upright orientations are stronger figural priors than are inverted or part-rearranged versions, at least for participants without brain damage.

However, studies involving participants with damage to the perirhinal cortex (PRC) of the medial temporal lobe (MTL) showed that familiar parts can function as figural priors: these participants perceived the figure equally often on the critical side of borders in displays depicting familiar configurations and novel part-rearranged configurations composed of upright parts (Barense et al., 2012). Later, using a masked priming paradigm in experiments conducted with participants without brain damage, Cacciamani et al. (2014) demonstrated that upright familiar parts activate representations of upright familiar wholes even when they are spatially rearranged into novel configurations. Barense et al. (2012) proposed that the intact PRC, known to discriminate between familiar versus novel configurations (Barense et al., 2007; Bussey et al., 2002; Miranda & Bekinschtein, 2018; Miyashita, 2019; O’Neil & Lee, 2019), modulates lower-level familiar part responses, reducing or removing their influence when present in novel configurations; PRC damage eliminates this modulation. Using fMRI (Cacciamani et al., 2017; Peterson et al., 2012, b) showed evidence consistent with this proposal.

In the present experiment, we examined how familiarity of parts and configurations fare when another figural prior—extremal edges—is present in the display.

### Extremal edges (EEs) in figure competition

Extremal Edges (EEs) are a special type of occlusion edge occurring when a 3D convex surface curves around to occlude a part of itself from the current viewing position (Ghose & Palmer, 2010; Huggins & Zucker, 2001a, 2001b; Palmer & Ghose, 2008). Adding a shading gradient to one side of a shared border introduces a strong EE figural prior when the equiluminant contours of the gradient are roughly parallel to the shared edge (Huggins et al., 2001; Palmer & Ghose, 2008). Equiluminant contours are the locus of same luminance levels. EEs are indicated by the steepest parts of the darker equiluminant portions of the gradient shown on the left side of the central border in Fig. 1c. It has been established that extremal edges (EEs) are strong figural priors even when an equally 3D convex volume with EEs orthogonal to the border lies on the opposite side of the border (Ghose & Palmer, 2016; Palmer & Ghose, 2008). In Fig. 1c, the shading gradients on both sides of the shared central border represent 2D projections of 3D cylinders with EEs either parallel or orthogonal to the shared border. For the shading gradient on the left, the “EE side,” the shared border is an extremal edge and is dominantly perceived as figural (83%). However, for the shading gradient on the right, the “non-EE side,” the shared border is not an extremal edge, the EEs are orthogonal to the shared border and it appears to be the ground side.

Ghose and Palmer (2010) showed that EEs tend to dominate figure assignment when the figural priors of smaller size, 2D-edge convexity, surroundedness, and familiar configuration (see Fig. 1d) lie on the opposite side of the shared border. In their experiments, they examined figure assignment in “congruent” and “incongruent” stimuli. In congruent stimuli, both EE and the other prior were present on the same side of the central border of a display with two regions on each side of the border (bipartite displays). In incongruent stimuli, EE and the other prior were present on the opposite sides of the central border. The congruent versus incongruent stimuli used in Ghose and Palmer (2010) were designed to examine figure assignment when figural priors cooperate versus compete, as described below.

### Competition in figure assignment

It is assumed that figure assignment is well described by cross-border inhibitory connections in interactive hierarchical models that take into account all relevant (inherently probabilistic) priors on both sides of shared borders (Craft et al., 2007; Froyen et al., 2010; Goldreich & Peterson, 2012; Kienker et al., 1986; Kogo & Ee, 2015; Vecera & O’Reilly, 1998). These models support the idea that figural priors present on both sides of a shared border engage in inhibitory competition to determine where the figure lies; the side that wins the competition is perceived as the “figure,” and the side that loses is perceived as the “ground.” For empirical support regarding these models, see Peterson et al. (2000), Peterson and Skow (2008), Salvagio et al. (2012), and Sanguinetti et al. (2016).

### Task set

Ghose and Palmer (2010) asked their participants which side of the shared border appeared to be nearer to them. Participants in their experiments reported the EE side as “nearer” on more than 90% of trials with both congruent (98%) and incongruent (92%) displays. This raises the question of whether the instructions to report “which side was nearer” introduced a task set that favored EE, a relative distance prior, over familiar configuration, a shape prior. It is becoming increasingly clear that instructions and task sets have a potent influence on perception because participants configure their perceptual/cognitive system to perform a specific task optimally and to preferentially perceive the stimulus attribute that is important for the given task (e.g., Ansorge & Neumann, 2005; Çukur et al., 2013; Harel et al., 2014; Monsell, 2003; Schneider & Logan, 2007; Sakai, 2008; Walther & Fei-Fei, 2007). However, it is not yet known whether instructions and task sets can alter the weights assigned to different types of figural priors. The current experiments were designed to investigate that question.

### Our study

In the present experiments, we seek to determine whether task set and instructions can influence the weights assigned to figural priors. Consider, for instance, displays used to investigate how EE (a relative distance prior) and familiar configuration (a shape prior) compete. Perhaps perceived shape and perceived distance dissociated such that while the EE side of the border appeared nearer, the familiar side was simultaneously perceived as shaped on a far plane. The two figural attributes of perceived shape and perceived depth ordering can dissociate across a shared border to produce the perception of shaped apertures (Bertamini & Croucher, 2003; Nelson & Palmer, 2001; Nelson et al., 2014; Palmer, Davis, Nelson, & Rock, 2008; Peterson, 2003). None of these studies exploring the perception of shaped apertures examined EE or competition between EE and familiar configuration. Nevertheless, they raise the possibility that if Ghose and Palmer’s instructions had emphasized the shaped attribute of figures rather than the near attribute, their results might have revealed evidence of a dissociation. In the present study, we used stimuli constructed such that EEs were present on the same side of the shared border as familiar configuration (congruent stimuli) or on the opposite side (incongruent stimuli; see Fig. 2) in three experiments. In Experiments 1 and 2, the task set was to report which side appeared to be shaped (in contrast to the task set to report which side is nearer used by Ghose and Palmer). In Experiment 2, additional instructions emphasized that some familiar objects might be present in the stimuli. In Experiment 3, both task set (to report the shaped versus the nearer side) and the presence versus absence of instructions emphasizing that familiar objects might be present were manipulated within subjects to allow a sensitive test of the role of task set and of instructions on figure assignment.

If the participants in Ghose and Palmer’s (2010) experiments had perceived shape on the familiar configuration side of the border at the same time they perceived the EE side as nearer, then the opposite pattern results would be expected when a “shaped-side” task set is used. That is, if the instructions were to emphasize shape, then we would expect participants to report perceiving the figure on the familiar configuration side of the central border on approximately the same percentage of trials on which Ghose and Palmer’s participants reported perceiving the figure on the EE side (~90% of trials). A finding such as this would require reevaluation of claims regarding the relative effectiveness of EE versus the familiar configuration prior and would suggest that the perceptual outcome for these incongruent displays was similar to that of a shaped aperture. A second possibility is that the “nearer-side” task Ghose and Palmer used operated to upweight the EE prior relative to the familiar configuration prior and “shaped-side” task might operate to upweight the familiar configuration, thereby increasing the likelihood that familiar configuration would win the competition with EE. In that case, we would expect participants to report perceiving the figure on the familiar configuration side of the central border on more than 10% of trials as they did under Ghose and Palmer’s instructions, but not on 90% of trials. Such results would provide empirical evidence for effects of task set in a previously unexamined realm—the weight assigned to relative distance versus shape priors competing to determine figure assignment. A third possibility is that the change in task set from highlighting the near attribute of figures to the shaped attribute will not substantially change figure assignment. In that case, we expect to obtain the same outcome Ghose and Palmer obtained when they pitted familiar configuration against EEs.

Our results show that participants performing the “shaped-side” task reported the EE-side as shaped less often in Experiments 1 and 2 than in previous experiments in which EE and familiar configuration were examined together and a “nearer-side” task was used. Experiment 2 showed that reports that the figure lay on the familiar configuration side of the border were further increased by instructions that familiar objects might be present. Experiments 1 and 2 allowed only between-subjects comparisons. In Experiment 3, where both task set and instructions that familiar objects might be present were manipulated within subjects, we replicated the effects of Experiment 2 and found effects of task set when EE and familiar configuration were incongruent. Thus, our results show that instructions and task set can influence the weighting of relative distance and shape priors for figure assignment. In addition, Experiments 2 and 3 showed that when instructions emphasized that familiar objects might be present, the influence of familiar parts emerged in participants with intact brains even though those parts were components of novel configurations. Finally, our results confirm previous evidence that EE is a strong figural prior, regardless of experiment instructions or task set.

## Experiment 1: Shaped-side task

In Experiment 1, the task instructions were changed to emphasize the shaped attribute of figures rather than the nearer attribute: Participants were asked to report which side of the border appeared to be shaped. Since familiar configuration is a shape prior, we reasoned that this change in task set might reveal effects of familiar configuration that were not evident when instructions emphasized nearness, which might have favored the relative distance prior, EE. We included inverted and part-rearranged versions of the familiar configuration as controls.

## Method

### Participants

A total of 39 participants (mean age = 22.7 years; 24 males and 15 females) was tested; 20 were undergraduate students at an American University, and 19 were students at a German University. All participants had normal or corrected-to-normal vision. They were naïve to the purpose and the nature of the experiment. They volunteered to participate for partial course credit in an undergraduate/graduate psychology course. They gave informed consent in accord with the policies of the Committee for the Protection of Human Subjects of the respective universities, which approved the experimental protocol.

We performed a post hoc power analysis using G*Power, which showed that the smallest possible effect size (partial eta squared) that can be detected with a sample size of 31 when power is fixed at 95% is 0.12.

### Apparatus

Displays were generated on a 14-inch Dell Notebook LCD (screen size 31 cm × 17.5 cm) with 1,280 × 800-pixel resolution in an otherwise dark room. The refresh rate of the screen was 60 Hz. The observer’s head was stabilized using a chin rest, and the screen was perpendicular to the line of sight. The size of the images was 4.34 cm × 5.79 cm (~ 4.3° × 5.8° at viewing distance of 57 cm). Presentation and response collection were controlled by a MATLAB program (The MathWorks Ltd.) using routines from the Psychophysics Toolbox (Brainard, 1997).

### Stimuli

The stimuli were created from a set of 27 black and white bipartite images divided into two equal area regions by a central border that represented a portion of a familiar (namable) object on one side in silhouette (stimuli used in Barense et al., 2012; Gibson & Peterson, 1994; Peterson et al., 2000; Peterson et al., 1998; Peterson & Gibson, 1993; see Fig. 2b). The objects portrayed by the silhouettes were identified correctly by at least 65% of pilot observers, indicating a good match to memory representations of known objects (see Peterson et al., 2000). We also included a matched condition, created by spatially rearranging the parts of the familiar configuration into a novel configuration (cf. Barense et al., 2012; Peterson et al., 1991; Peterson et al., 1998; Peterson et al., 2000). Parts were defined between two successive concave cusps identified from the side of the central border where the familiar configuration lay. Based on pilot results provided by Peterson et al. (2000), the part-rearranged silhouettes failed to provide a good match to memory representation of known objects (cf. Flowers et al., 2020).

These basic shapes were used to extract the coordinates of the shared edge, which was then used for rendering a surface of revolution (SOR) using POVRAY (an open-source ray-tracing program). SOR is a surface created by rotating a given 2D curve (“generatrix”) around a straight axis (see Fig. 2a). SORs are vase-like shapes with profile determined by the generatrix. Each cut through the surface perpendicular to the axis is circular and therefore convex at every tangent point. When SORs are orthographically projected to a viewpoint at the center of the axis, the projected edge produces an extremal edge (EE) with a shape equivalent to the generatrix. The surfaces of the rendered objects were primarily Lambertian surfaces with the following parameters in POVRAY: diffuse 0.75, phong 0.15, phong size 20. Then, the SORs were cropped along the mid-axis, resulting in the final stimuli that had the same dimensions as the original 27 black and white images. For every generatrix, two SORs were rendered by choosing different axes of rotation to generate “congruent” and “incongruent” versions. In the congruent version, both familiar configuration and EE favored the same side of the bipartite image as the figure. In the incongruent version, one side of the bipartite image was favored as the figure by EE, whereas the other side was favored as the figure by familiar configuration.

### Design

A complete set of 216 displays was generated by a four-way factorial within-subjects design (see Fig. 2b). The first factor was orientation: upright or inverted. The second factor was the configuration type suggested by the shared edge: intact familiar configuration or part-rearranged (PR) novel configuration. The third factor was the congruency of the two priors—familiar configuration and EE: congruent or incongruent. In the congruent condition, both EE and familiar configuration favored the same side of the bipartite image as the figure, whereas in the incongruent condition, they favored opposite sides. The fourth factor was left/right side of the familiar configuration in each of the 27 basic images from Peterson and Gibson (1994a, 1994b).

Five of the original images (stop sign, cone, hydrant, hand, pine tree) and their variants were used for the practice block (*N* = 20 stimuli). The remaining 22 images were divided into two groups such that intact familiar configuration versions of 11 stimuli (lamp, milk can, pear, pineapple, seahorse, snowman, toilet, umbrella, wineglass, woman, wrench) were shown in Block-i and their PR versions were shown in Block-ii. For the other 11 stimuli (arrow, apple, bell, bulb, coffee pot, cow, tree, face, letter *F*, guitar, house) the PR version was shown in Block i and the intact version was shown in Block ii (the order of Blocks i and ii was counterbalanced across participants). Thus, half the stimuli were viewed first in their intact version; the other half were viewed first in their PR version. Both upright and inverted versions of either intact or PR configurations were presented in a single block. In each block trials were presented in random order.

### Procedure

**Fig. 3:**
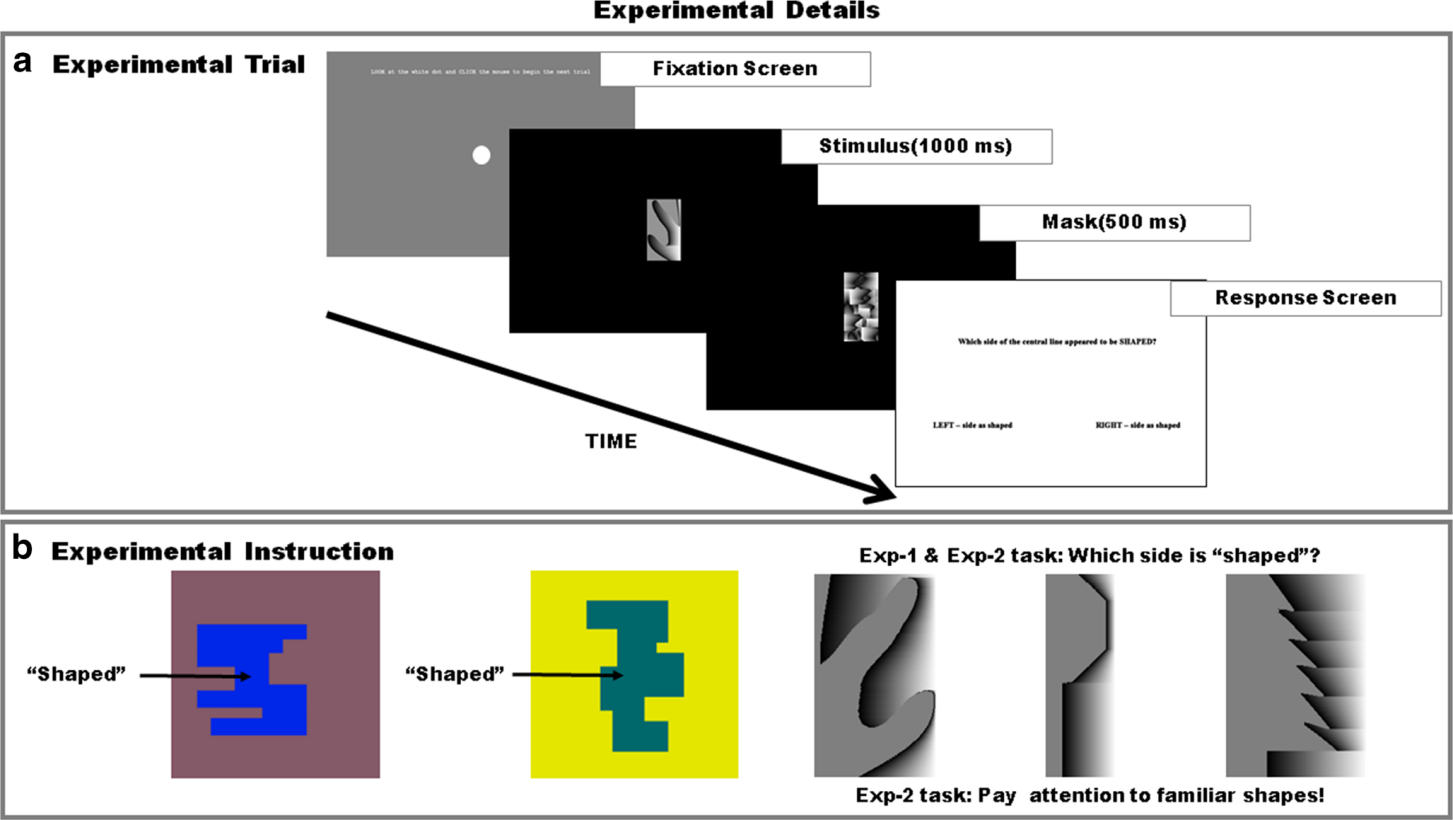
**a** Experimental trial. The trial structure illustrated here was used for both Experiments 1 and 2. **b** Shaped versus shapeless. Shape judgment task was explained by indicating that blue and green regions appear to have definite “shape” followed by clarification that stimuli will be bipartite images. **c** Task instruction. Experiment 1: Shaped-side task instruction “Your task will be to judge which side of the shared border is ‘shaped.’ Please report your first impression/ gut reaction.” Experiment 2: Shaped-side task with familiarity instruction present “Your task will be to judge which side of the shared border is ‘shaped.’ Sometimes the shape can be a familiar shape. PAY ATTENTION. Please report your first impression/ gut reaction. Verbally identify the familiar shapes shown here.” Sample images from the practice session are illustrated

A schematic illustration of the trial structure is shown in Fig. 3a. The task of “shape judgment” was explained to the participants by using the illustrations shown in Fig. 3b. They were instructed that “the green and the blue regions in the two pictures, appear to have a definite ‘shape’ while the yellow and the purple regions appear to be ‘not shaped’ and are seen as backgrounds.” Participants were further instructed that the stimuli for this experiment consist of images with two regions that share a border. They were shown the images in Fig. 3c as examples. These were images in which the familiar configuration was a portion of a hand, a stop sign, and a pine tree (three of the practice stimuli) in the familiarity/EE incongruent condition. Then the task was explained with the following statements: “Your task will be to judge which region is ‘shaped.’ There is no correct answer, please report your first impression and enter your response with a mouse click.” Participants were reminded to report their first impression in a second sentence where the term “gut reaction” was also used.

A white fixation dot appeared at the beginning of each trial. The observers were asked to fixate on the white dot so that they were looking directly at the shared contour when the stimulus appeared. After the observers had fixated on the white dot, they clicked the mouse to see a bipartite stimulus. The stimulus was exposed for 1,000 ms, followed by a mask for 500 ms, and then by a response screen. The response screen displayed the task instruction “Which side of the central line appeared to be SHAPED?” and two response boxes labeled “left” and “right,” which could be selected with a mouse click. The fixation screen reappeared after the observer provided their response. There was no time-out. The practice block and the two experimental blocks took approximately 30 minutes for completion.

### Results and discussion

**Fig. 4:**
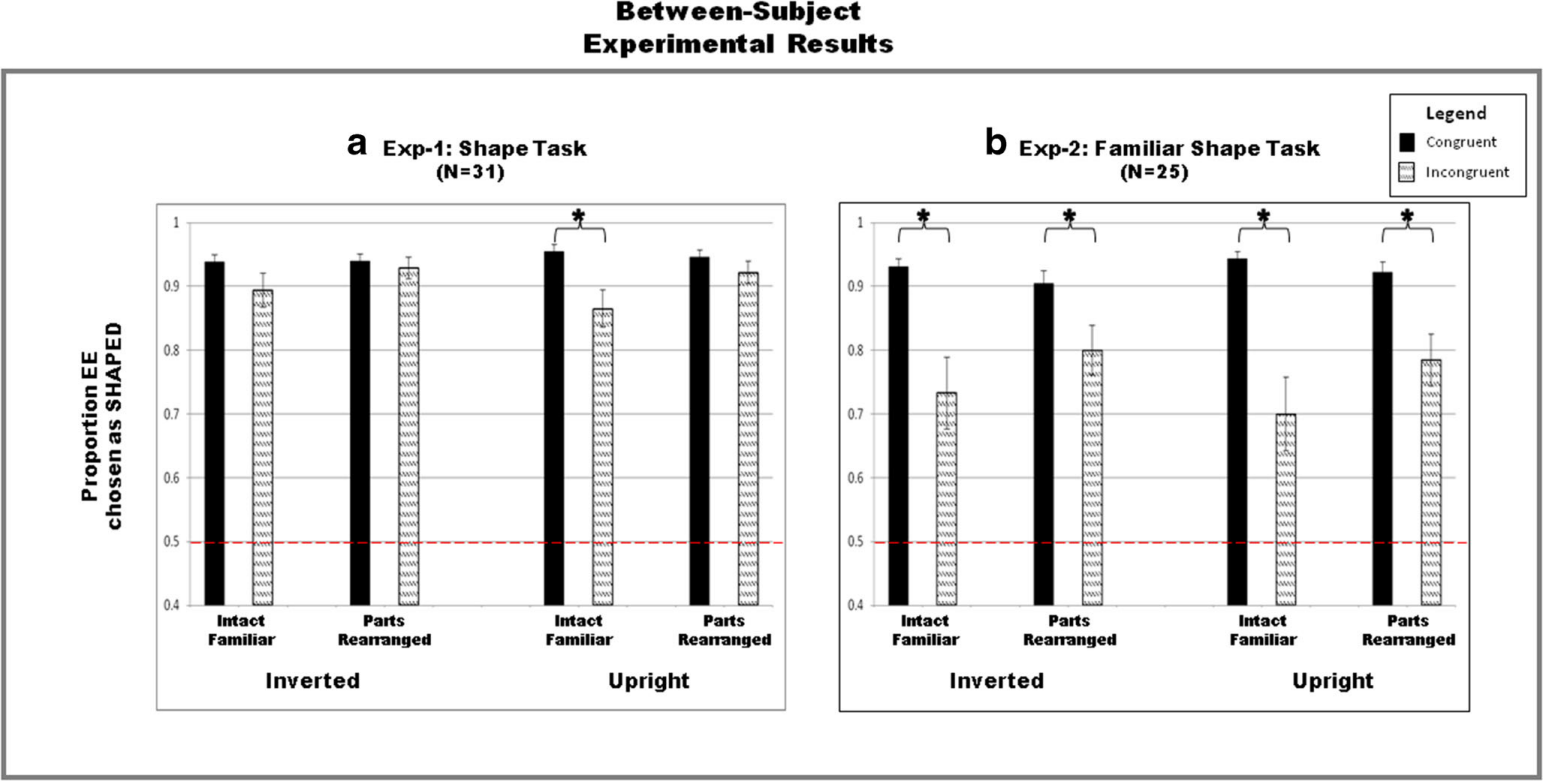
Results. Data are plotted as percentage of trials the EE side was chosen as shaped. **a** Experiment 1. Familiarity instruction absent, shaped-side task. **b** Experiment 2. Familiarity instruction present, shaped-side task. The data show that the closer EE side also appears to be shaped unless familiarity is “primed” with instructions. Error bars correspond to the standard errors of the means

The data were coded as the percentage of trials on which the EE side was chosen as “shaped” (see Fig. 4a). Based on an outlier analysis, eight participants were removed from the final analysis, because their data for one or more conditions were below two standard deviations from the mean, a standard criterion^1^ used in our labs. The results are based on 31 participants.

There was no main effect of left/right position of the EE side (*p* > .05 for both factors). The EE side was chosen as shaped on 92% of the trials compared with the non-EE side. Reports of the EE side as shaped were subjected to a 2 × 2 × 2 repeated-measures analysis of variance (ANOVA) with three factors (orientation: inverted/upright; configuration type: intact/PR; and EE/Familiarity congruence: incongruent/congruent). A significant main effect of congruence was observed, *F*(1, 30) = 5.38, *p* = .027, η_p_^2^ = 0.15: Mean reports of the EE side as the shaped side were lower when EE and familiarity were incongruent rather than congruent (incongruent: 90% vs. congruent: 95%), showing that familiar configuration modulated reports of perceived shape. Similarly, a small but significant main effect of familiar configuration type was observed, *F*(1, 30) = 7.55, *p* = .010, η_p_^2^ = 0.20: Mean EE-side responses were lower when the familiar configuration was intact versus part-rearranged (intact: 91% vs. PR: 93%). Effects of familiar configuration were largest in the upright incongruent condition: As can be seen in Fig. 4, for the upright incongruent displays in which the familiar configuration was intact, the reports of the shape on the EE side of the border were reduced (86%) relative to the congruent displays (95%), *t*(30) = 3.21, *p =* .003. This difference was not observed for PR configurations in upright displays (92% vs. 94%), nor was it observed for inverted displays (intact: 89% vs. 94%; PR: 93% vs. 94%), *p*s > .06. This effect was shown to be statistically significant by a three-way interaction among orientation, configuration type, and congruency, *F*(1, 30) = 5.55, *p* = 0.025, η_p_^2^ = 0.16. The three-way interaction subsumed two-way interactions between orientation and congruency, *F*(1, 30) = 12.98, *p* = .001, η_p_^2^ = 0.30, as well as between configuration type and congruency, *F*(1, 30) = 6.38, *p* = 0.017, η_p_^2^ = 0.18. Neither a significant main effect of orientation was observed, *F*(1, 30) = 0.40, *p* = .530, η_p_^2^ = 0.01, nor did orientation interact with configuration type, *F*(1, 30) = 0.35, *p* = .558, η_p_^2^ = 0.01.

These results demonstrate that the change in task set had a small but significant effect on the pattern of the results. The EE side of the border was perceived as shaped on only 86% of trials in the incongruent condition when the region on the opposite side of the central border suggested an upright intact familiar configuration, compared with 95% of trials in the congruent condition when EE and familiarity favored the same side as figure. An effect of task set was evident in comparison with nearer reports published by Ghose and Palmer (2010): 92% versus 98% for incongruent versus congruent conditions, respectively. These results suggest that the weight placed on the familiar configuration prior is increased when task instructions emphasize shape rather than nearness. The results of this experiment provide empirical evidence that task set can affect the relative weighting of depth versus shape figural priors that engaged in inhibitory competition for figure assignment outside of awareness. Note, however, that a statistical analysis could not be conducted because more and different stimuli were used in the present experiment than in Ghose and Palmer’s experiment using a near-side task set. Despite suggesting that task set affects response, the results of Experiment 1 also confirm that EE is a more effective figural prior than familiar configuration. Moreover, the results provide no evidence that shape and nearness were dissociated in Ghose and Palmer’s previous experiments. Had they been dissociated, as they are when shaped apertures are perceived, the figure would have been reported on the familiar configuration side of the border on the majority of trials, and it was not.

## Experiment 2: Shaped-side task with instructions regarding the potential presence of familiar objects

In Experiment 1, the influence of task set was evident, albeit small. The goal of Experiment 2 was to investigate whether the weighting of the familiar configuration prior can be increased further by instructions informing participants of the potential presence of familiar objects. In this experiment, in addition to asking the participants to report which side of the shared border appeared to be shaped, the instructions stated that some familiar objects might be present in the stimuli. The apparatus, stimuli, design, and other aspects of the procedure were exactly the same as in Experiment 1.

## Method

### Participants

A total of 29 participants was tested (mean age = 25 years, 11 males and 19 females); 10 were undergraduate students at an American University, and 19 were students at a German University. There was no overlap between the groups of participants tested in Experiments 1 and 2. All participants had normal or corrected-to-normal vision. They were naïve to the purpose and the nature of the experiment. They volunteered to participate for partial course credit in an undergraduate/graduate psychology course. They gave informed consent in accord with the policies of respective Committees for the Protection of Human Subjects, which approved the experimental protocol.

We performed a post hoc power analysis using G*Power, which showed that the smallest possible effect size (partial eta squared) that can be detected with a sample size of 25 when power is fixed at 95% is 0.14.

### Apparatus

The apparatus were the same as in Experiment 1.

### Stimuli

Stimuli were the same as in Experiment 1.

### Design

The design was the same as in Experiment 1.

### Procedure

The procedure was the same as in Experiment 1, except for a minor difference in the instructions. In addition to the “shaped side” task set from Experiment 1, the following lines were added: “Sometimes the shape can be a familiar shape. PAY ATTENTION.” In addition, on the screen where the sample images of a hand, stop sign, and pine tree in the incongruent condition were shown, the instruction was modified to “Some examples of images are shown below; do you see any familiar shapes?” After reading this instruction, the experimenter paused, and participants were required to verbally identify the familiar shapes illustrated in these images. Finally, after the task instructions that explained the left/right response, it was added that “There is no ‘correct answer’; please use your first impression (modified by the term ‘gut reaction’ in a repetition of this phrase), and please pay attention to familiar shapes.”

### Results and discussion

As in Experiment 1, the data were coded as the percentage of trials on which the EE side was chosen as shaped (see Fig. 4b). Based on an outlier analysis, four participants were removed from the final analysis because their data for one or more conditions were more than two standard deviations from the mean. The results are based on 25 participants.

There was no main effect of left/right position of the EE side (*p* > .05 for both factors).

The EE side was chosen as shaped on 84% of the trials compared with the non-EE side. A 2 × 2 × 2 repeated-measures ANOVA with three factors (orientation: inverted/upright; configuration type: intact/PR; and EE/Familiarity congruence: incongruent/congruent) showed a significant main effect of congruency, *F*(1, 24) = 18.88, *p* = .000, η_p_^2^ = 0.44: Mean reports that the EE side of the border was shaped were higher for congruent compared with incongruent conditions (congruent: 93% vs. incongruent: 75%). This difference of 18 percentage points is greater than that in Experiment 1 (9 percentage points), providing further evidence that relative weights assigned to figural priors are regulated by instructions. This effect was modulated by configuration type and congruency, *F*(1, 24) = 12.61, *p* = .002, η_p_^2^ = 0.34. A two-way interaction between orientation and congruency, *F*(1, 24) = 6.21, *p* = .020, η_p_^2^ = 0.21, revealed that there was a larger reduction in reports of the EE side as shaped in the incongruent condition compared with the congruent condition for upright displays (congruent: 94% vs. incongruent: 70%) than inverted displays (congruent: 93% vs. incongruent: 73%). Again, there was a small but significant effect of configuration type, *F*(1, 24) = 6.25, *p* = .020, η_p_^2^ = 0.20: Mean reports of the figure on the EE side of the border were lower when an intact upright familiar configuration rather than a PR novel configuration was sketched on the opposite side of the border (intact: 83% vs. PR: 85%). Nevertheless, the decrease in percentage for EE figural reports between congruent and incongruent conditions was statistically significant for all familiar configurations: upright intact, *t*(24) = 4.55, *p <* .0001; inverted intact, *t*(24) = 3.88, *p =* .001; upright PR, *t*(24) = 4.45, *p <* .0001; and inverted PR, *t*(24) = 3.37, *p =* .002. This adds to the evidence that familiar parts in addition to intact familiar configurations can participate in cross-border competition for figural status (e.g., Barense et al., 2012), shown here for the first time in participants without damage to the PRC of the MTL. Again, no significant main effect of orientation was obtained, *F*(1, 24) = 0.81, *p* = .376, η_p_^2^ = 0.03. The three-way interaction among orientation, configuration type, and congruency was not statistically significant, *F*(1, 24) = 0.33, *p* = .571, η_p_^2^ = 0.01, nor was the two-way interaction between orientation and familiar configuration, *F*(1, 24) = 0.77, *p* = .389, η_p_^2^ = 0.03.

The Experiment 2 results show that when attention is drawn to the potential presence of familiar objects, the shaped entity is more likely to be perceived on the familiar side of the border on incongruent trials when the EE prior and the familiarity prior compete than on congruent trials when the two figural priors cooperate. This is especially true when the displays are upright, but, unlike in Experiment 1, it is also true when the displays are inverted. Thus, when task instructions draw attention to familiarity, the effects of familiarity are enhanced for both upright and inverted familiar configurations and for upright and inverted PR novel configurations. Thus, the enhanced shape instructions in Experiment 2 where participants were instructed to pay attention to the possible presence of familiar objects up-regulated effects of both familiar parts and familiar configurations on figure assignment. Note that these results cannot be explained by a strategy to look for familiar shapes for two reasons: First, the PR configurations are perceived as novel configurations rather than as familiar objects (Flowers et al., 2020; Peterson et al., 1991; Peterson et al., 1998; Peterson et al., 2000). Second, participants were instructed to report their first impression.

Additionally, even though the effect of familiarity was enhanced on incongruent compared with congruent trials in Experiment 2, the EE-side was perceived as the shaped side on an average of 75% of the trials with incongruent displays, which is significantly greater than expected on the basis of chance, *t*(99) = 10.34, *p* < .001.

### Between-experiment comparison

A between-subjects ANOVA was conducted on reports of the EE side as shaped in Experiments 1 and 2, with orientation, configuration type, and congruency as within-subjects factors. The results showed a significant interaction between experiment and congruency, *F*(1, 54) = 10.03, *p* = .003, η_p_^2^ = 0.16, confirming that when the instructions emphasized the potential presence of familiar objects in Experiment 2, the figure was more likely to be perceived on the side of the border of incongruent displays, where intact as well as PR novel configurations lay, than in Experiment 1, where instructions did not emphasize the possible presence of familiar objects. This effect did not vary with orientation, *F*(1, 54) = 0.017, *p* = .898, η_p_^2^ = 0.00. No other main effects or interactions were significant, *p*s > .144.

## Experiment 3: Within-subjects manipulations of task set and instructions regarding the potential presence of familiar objects

**Fig. 5:**
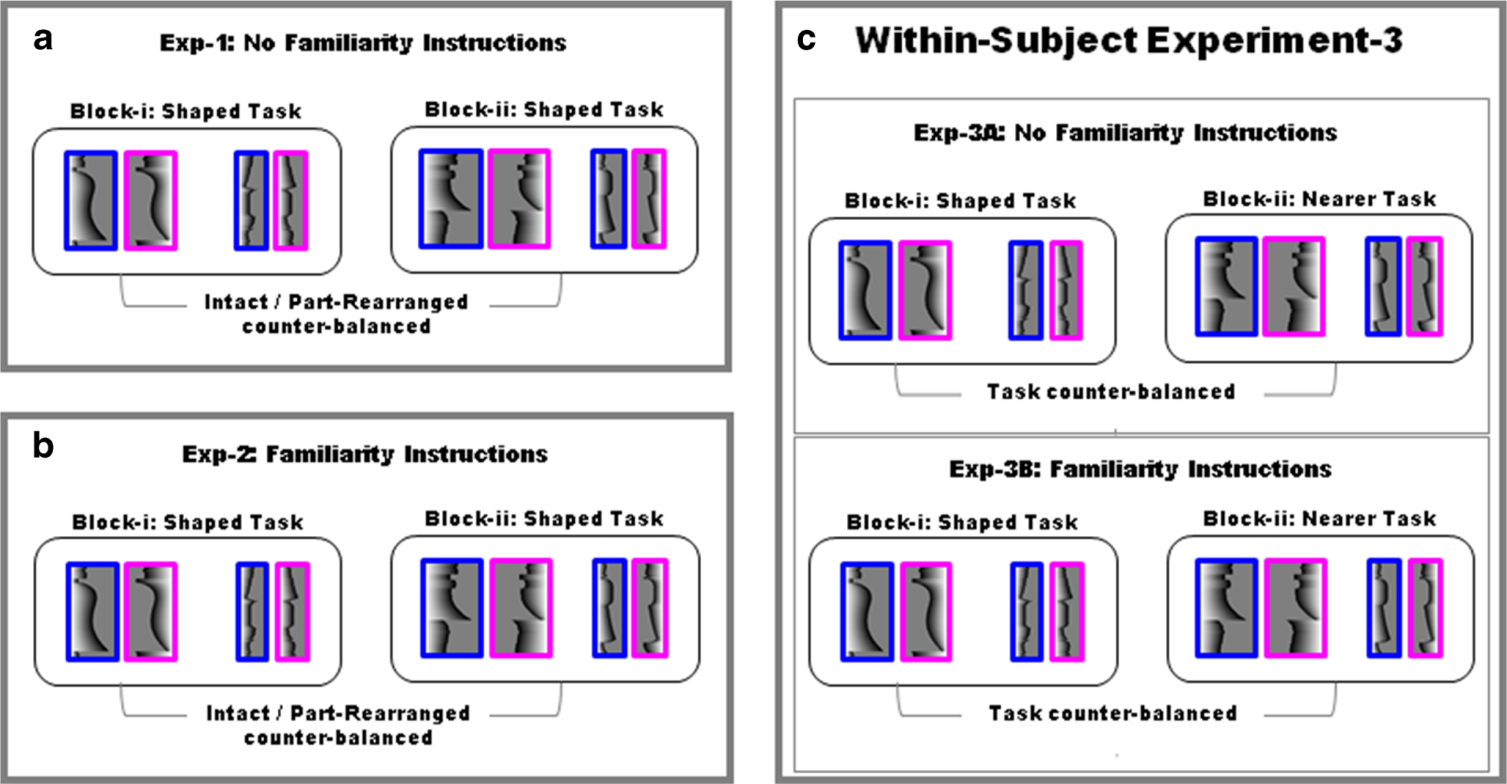
Illustration of difference in design between Experiments 1, 2 (between-subject), and 3 (within-subject). Experiments 1 and 2 involved shaped-side task only, while Experiment 3 included two task sets: shaped-side task and nearer-side task, with task order counterbalanced across participants. Experiment 3b always followed Experiment 3a

In Experiment 3, task set was directly manipulated. In a within-subjects design, the participants performed two different tasks—namely, “which side appears *shaped t*o you?” and “which side appears *nearer* to you?” in two counterbalanced subblocks within each half of the experiment. In the first half of Experiment 3, instructions regarding the potential presence of familiar objects were absent (henceforth, Experiment 3a). In the second half of Experiment 3, the instructions regarding the potential presence of familiar objects were present (henceforth, Experiment 3b). The order of the absence/presence of the “potential familiar object” instructions could not be counterbalanced because (a) we are attempting to replicate Experiment 1 in Experiment 3a, where instructions regarding the potential presence of familiar objects were not given, and (2) we did not consider it feasible to ask participants to disregard the potential presence of familiar objects once they knew they might be present. Except for the manipulation of task set, Experiment 3a was identical to Experiment 1 because both were marked by the absence of “potential familiar object” instructions, and Experiment 3b was identical to Experiment 2 because both were marked by the presence of “potential familiar object” instructions. Thus, except for the within-subjects manipulation of task set, Experiment 3a is a replication of Experiment 1 and Experiment 3b is a replication of Experiment 2 (see Fig. 5c). The apparatus and stimuli were exactly the same as in Experiment 1.

## Method

### Participants

A total of 29 participants was tested (mean age = 25.9 years, 20 males and nine females); all were students at a German University. All participants had normal or corrected-to-normal vision. They were naïve to the purpose and the nature of the experiment. They volunteered to participate for partial course credit in an undergraduate/graduate psychology course. They gave informed consent in accord with the policies of the Committee for the Protection of Human Subjects, which approved the experimental protocol.

We performed a post hoc power analysis using G*Power, which showed that the smallest possible effect size (partial eta squared) that can be detected with a sample size of 24 when power is fixed at 95% is 0.15.

### Apparatus

The apparatus were the same as in Experiment 1.

### Stimuli

Th stimuli were the same as in Experiment 1.

### Design

The experiment was a within-subjects design with two parts corresponding to Experiments 1 and 2, described above, marked by the absence or presence of instructions regarding the potential presence of familiar objects. For the ease of nomenclature, we label these blocks as Experiment 3a and Experiment 3b, respectively. Within Experiments 3a and 3b, there were two subblocks corresponding in design to those described in Experiment 1, for division of the 216 bipartite images. In Experiment 3, unlike Experiments 1 and 2, participants were given different task sets (“which side appears to be shaped?” and “which side appears to be nearer?”) in the two subblocks, with task set order counterbalanced across observers (see Fig. 5c).

### Procedure

The procedure was the same as in the previous experiments, until the participants were shown the bipartite stimuli. Here, they were informed that they would be performing either a “shape judgment task” or a “nearness judgment task” in four blocks. Each of these blocks was preceded by a short practice block to familiarize the participant with the task for the upcoming experimental block. The practice stimulus set was the same as in Experiments 1 and 2. Except for the added manipulation of task set, the procedure for Experiment 3a was exactly the same as that of Experiment 1, and the procedure for Experiment 3b was the same as that of Experiment 2 (i.e., the instructions regarding the potential presence of familiar objects were given). In both Experiments 3a and 3b, participants were instructed to report their first impression. This was stressed through repetition, and when it was repeated, “gut reaction” was added after “first impression” for emphasis. At the end of the experiment, participants filled out a questionnaire to report the percentage of trials on which they did not report their first impression (gut reaction). We planned to eliminate participants who responded that they did not report their first impression on >20% of trials.

### Results and discussion

**Fig. 6:**
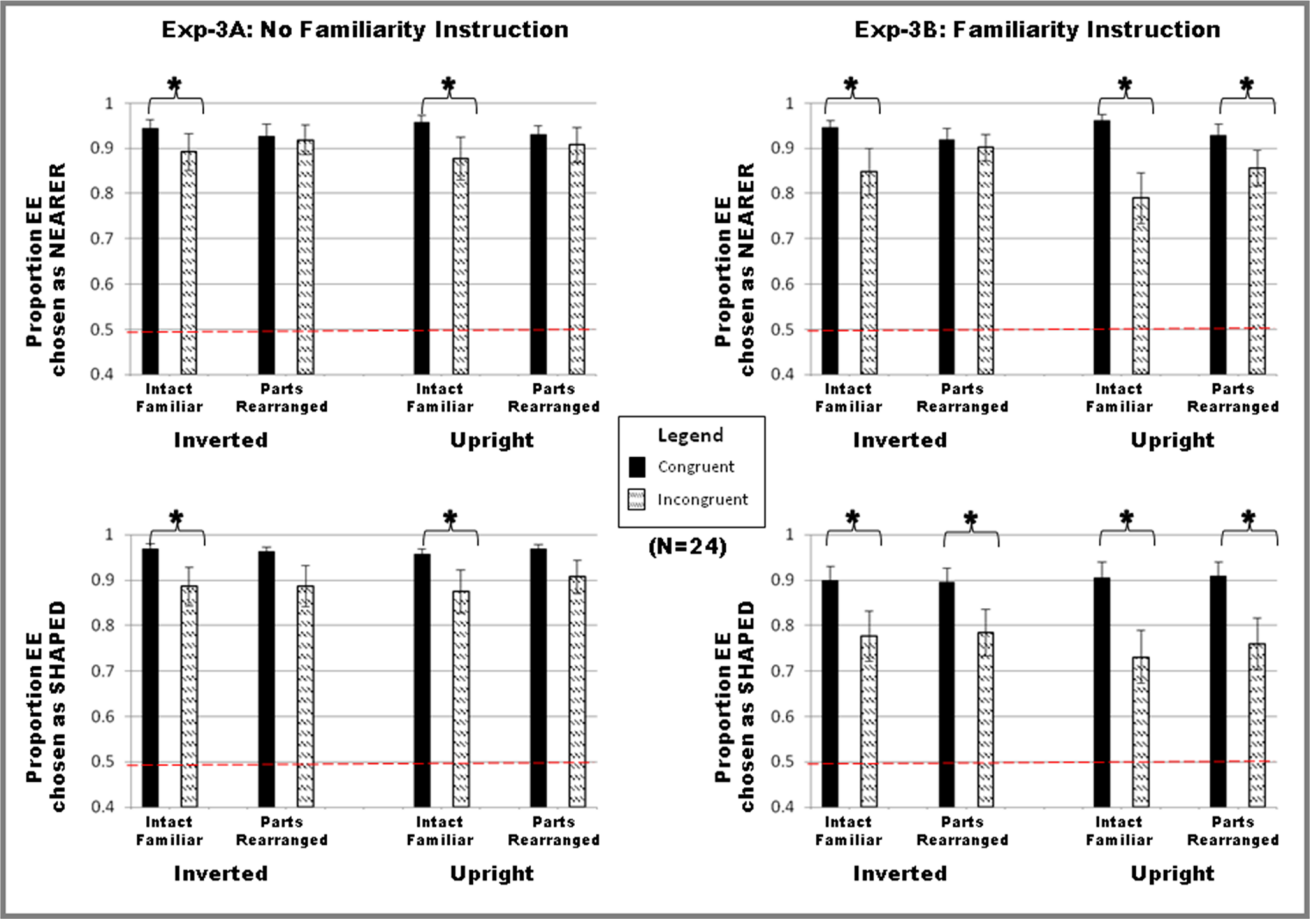
Results. Data are plotted as percentage of trials the EE side was chosen as nearer (top row) and shaped (bottom row) for Experiment 3a: familiarity instruction absent and Experiment 3b: familiarity instruction present. The data show that the closer EE side also appears to be shaped unless familiarity is “primed” with instructions emphasizing its presence in the stimuli. Error bars correspond to the standard errors of the means

The data were coded as the percentage of trials on which the EE side was chosen as “shaped” and “nearer” (see Fig. 6). Based on an outlier analysis, five participants were removed from the final analysis because their data for one or more conditions were below two standard deviations from the mean. No participants were excluded for not reporting their first impression. The results are based on 24 participants.

There was no main effect of left/right position of the EE side (*p* > .05 for both factors).

Nor was there a main effect of the between subjects factor of task set order (*p* > .05). Overall, the EE side was chosen as the “shaped side” on 88% of trials and as the “nearer side” on 91% of trials compared with the non-EE side. Reports of the EE side as figure were subjected to a 2 × 2 × 2 × 2 ×2 repeated-measures ANOVA, with five factors (potential familiar object instruction: present/absent (i.e., Experiments 3a–b); task set: shaped/nearer side; orientation: inverted/upright; configuration type: intact/PR; and EE/Familiar configuration congruence: incongruent/congruent). The main effect of task set (shaped-side vs. near-side) did not reach significance, *F*(1, 23) = 1.85, *p* = 0.188, η_p_^2^ = 0.07. A significant main effect of potential familiar object instruction was observed, *F*(1, 23) = 6.22, *p* = .020, η_p_^2^ = 0.21: Mean reports of the EE side as the shaped or nearer side were higher in Experiment 3a where the potential familiar object instruction was absent compared with Experiment 3b (3a: 92% vs. 3b: 86%), showing that instructions regarding the potential presence of familiar objects in Experiment 3b upweighted the effects of the figural prior of familiar configuration in the competition for figural assignment and replicating the difference observed in the comparison of Experiments 1 and 2. A small but significant main effect of orientation was observed, *F*(1, 23) = 8.29, *p* = .008, η_p_^2^ = 0.27: Mean reports of the EE side as the figural side were higher when paired with inverted familiar configurations compared with upright ones in incongruent displays (inverted: 90% vs. upright: 89%). These results are consistent with previous explanations that memory representations activated by upright familiar configurations reach threshold faster than those activated by inverted ones, and consequently exert a greater influence in the competition for figural assignment. A significant main effect of congruence was observed, *F*(1, 23) = 9.62, *p* = .005, η_p_^2^ = 0.30: Mean reports of the EE side as the figural side were higher when EE and familiarity were congruent rather than incongruent (congruent: 94% vs. incongruent: 85%), showing that familiarity modulated reports of perceived figural attributes. There was a statistically significant three-way interaction among potential familiar object instruction, orientation, and congruency, *F*(1, 23) = 11.87, *p* = .002; η_p_^2^ = 0.34. The difference between congruent and incongruent EE-side responses was larger in upright than in inverted displays (difference = 14% and 9%, respectively, *p* < .002) in Experiment 3 when the potential of familiar objects instruction was present. The difference between congruent and incongruent EE-side responses were significant (both = 5%, *p*s < 0.04), but did not differ for upright and inverted displays in Experiment 3a when the potential of familiar objects instruction was absent, and these differences were smaller than in Experiment 3b. The three-way interaction subsumed significant two-way interactions between potential familiar object instruction and orientation, *F*(1, 23) = 6.88, *p* = .015, η_p_^2^ = 0.23, potential familiar object instruction and congruency, *F*(1, 23) = 6.95, *p* = .015, η_p_^2^ = 0.23, and orientation and congruency, *F*(1, 23) = 9.01, *p* = .006, η_p_^2^ = 0.28. These results support the hypothesis that, when given instructions regarding the potential presence of familiar objects in Experiment 3b, participants upweighted familiarity as a figural prior, and this upweighting extended to familiar parts as well as familiar configurations replicating the pattern observed in Experiment 2.

An effect of task set was evident in a significant interaction between task set and congruency, *F*(1, 23) = 8.80, *p* = .007, η_p_^2^ = 0.28, and a marginally significant three-way interaction between task set, configuration type, and congruency, *F*(1, 23) = 3.91, *p* = .06, η_p_^2^ = 0.15. The EE responses showed a greater difference between the congruent and the incongruent versions in the “shaped-side” task (congruent: 93% vs. incongruent: 83%), *t*(23) = 3.16, *p =* .004, than in the “nearer-side” task (congruent: 94% vs. incongruent: 87%), *t*(23) = 2.92, *p =* .008. The mean difference between EE responses for the “shaped side” versus “nearer side” task was significant for the incongruent condition (shaped: 83% vs. nearer: 87%), *t*(23) = −2.1, *p =* .047, but not for the congruent condition (shaped: 93% vs. nearer: 94%). These results support the claim regarding task set based on the results of Experiments 1 and 2 that the differential weighing of figural priors is evident for the shaped-side task when EEs and familiar configuration, a shape prior, compete for figural assignment, although EEs remain a dominant figural prior.

In order to better understand the influence of the information regarding the potential presence of familiar objects and the extent to which the results of Experiment 3 replicate those of Experiments 1 and 2, the results of Experiments 3a and 3b were subjected to separate 2 × 2 × 2 × 2 repeated-measures ANOVAs with four factors (task set: shaped/nearer; orientation: inverted/upright; configuration type: intact/PR; and EE/Familiarity congruence: incongruent/congruent).

#### Experiment 3a

A significant main effect of congruence was observed, *F*(1, 23) = 5.14, *p* = .033, η_p_^2^ = 0.18: Mean reports of the EE side as the shaped/nearer side were higher when EE and familiarity were congruent rather than incongruent (congruent: 95% vs. incongruent: 89%), showing that familiar configuration modulated reports of perceived figure assignment. There was a significant two-way interaction between configuration type and congruency, *F*(1, 23) = 5.86, *p* = .024, η_p_^2^ = 0.20: The difference between congruent and incongruent displays was greater for intact familiar configurations (congruent: 96% vs. incongruent: 88%) than for PR configurations (congruent: 95% vs. incongruent: 91%). This effect is similar to that observed in Experiment 1, except that the difference between inverted congruent and incongruent stimuli did not reach significance in Experiment 1. Neither the main effect of task set, *F*(1, 23) = 0.13, *p* = .72, η_p_^2^ = 0.005, nor the interaction between task set and congruency was statistically significant, *F*(1, 23) = 2.78, *p* = .11, η_p_^2^ = 0.11, in Experiment 3a. Thus, without instructions regarding the potential presence of familiar objects the “shaped-side” task set alone was insufficient to increase the weight of the figural prior of familiar configuration, at least when task set was manipulated within subjects who were initially instructed about both the shaped-side and the nearer-side task sets.

#### Experiment 3b

A significant main effect of congruence was observed, *F*(1, 23) = 11.63, *p* = .002, η_p_^2^ = 0.34: Mean reports of the EE side as the shaped or nearer side were higher when EE and familiarity were congruent rather than incongruent (congruent: 92% vs. incongruent: 81%), showing that familiarity modulated perceived figure reports. A small but significant main effect of orientation was observed, *F*(1, 23) = 9.86, *p* = .005, η_p_^2^ = 0.3: Mean reports of the EE side as the shaped or nearer side were higher when EE was paired with inverted familiar configurations compared with upright ones (inverted: 87% vs. upright: 86%), again consistent with the proposal that upright familiar configurations activate previous experience faster than inverted ones. There was a significant two-way interaction between task set and congruency, *F*(1, 23) = 9.5, *p* = .005, η_p_^2^ = 0.29: The EE responses for the shaped-side task showed a greater difference between the congruent and the incongruent versions (shaped congruent: 90% vs. incongruent: 76%), *t*(23) = 3.52, *p =* .002, compared with the nearer-side task (nearer congruent: 94% vs. incongruent: 85%), *t*(23) = 3.13, *p =* .005. The mean difference between EE responses for shaped versus nearer task was significant for the incongruent condition (76% vs. 85%), *t*(23) = −2.54, *p =* .018, but not for the congruent condition, indicating an effect of task set when a relative distance prior (EE) competes with a relative shape prior (familiarity). This interaction indicates that although EEs are a strong relative distance figural prior, the influence of familiarity, a shape prior, can be upweighted by experiment instructions so that it competes more strongly for figural assignment, replicating the results of Experiment 2. This two-way interaction subsumes a marginally significant main effect of task set, *F*(1, 23) = 3.72, *p* = .066, η_p_^2^ = 0.14, wherein the EE side was chosen as nearer more often than it was chosen as shaped (nearer: 89% vs. shaped: 83%). In addition, there was a significant two-way interaction between orientation and congruency, *F*(1, 23) = 13.09, *p* = .001, η_p_^2^ = 0.36. The difference between congruent and incongruent displays was greater for upright (congruent: 93% vs. incongruent: 78%) than for inverted (92% vs. 83%) displays; and a significant two-way interaction between configuration type and congruency, *F*(1, 23) = 5.62, *p* = .027, η_p_^2^ = 0.20. The difference between congruent and incongruent displays were greater for intact familiar configurations (congruent: 93% vs. incongruent: 79%) than for part-rearranged configurations (91% vs. 83%). These results show that, although the instructions to pay attention to the possibility of familiar displays upweighted familiar parts as well as familiar configurations, as observed in Experiment 2, the effects of upright intact familiar configurations remained larger than effects of inverted or PR configurations.

## Discussion

Instructions given at the beginning of experiments have been shown to influence interactions between brain signals from higher-order cognitive areas and lower-order sensory areas (Çukur et al., 2013; Harel et al., 2014; Sakai, 2008) as well as behavioral outcomes (e.g., Ansorge & Neumann, 2005; Kiefer, 2012; Schneider & Logan, 2007; Walther & Fei-Fei, 2007). Using transcranial magnetic stimulation (TMS), Nakamura et al. (2006) showed that information about the stimuli is conveyed via different routes depending on the task set and that this differential routing occurs outside of participants’ conscious awareness.

In figure–ground perception, two sides sharing a border compete for border ownership. The side that wins is perceived as the figure with two attributes—it appears shaped and nearer than the other side of the border. It was long thought that figure–ground resolution was preattentive (Lazareva et al., 2006; Lester et al., 2009; Nelson & Palmer, 2007; Wong & Weisstein, 1982), but recent research has challenged that view (for review, see Peterson, 2019). Here, we investigated whether the weight placed on two figural priors can be changed by instructions. In the three experiments reported in this paper, two figural priors, EE and familiar configuration, were placed in competition and cooperation across a shared border in carefully constructed stimuli. In a task using “which side is nearer” instructions, Ghose and Palmer (2010) had established EE as a strong figural prior that dominates familiar configuration (as well as border convexity, smaller size, and surroundedness). By definition, EE is a relative distance prior, whereas familiar configuration is a shape prior; hence, instructions to report which side appeared to be nearer may have favored EE. Therefore, in this study, we examined whether instructions to report which side appeared to be shaped altered the perceived figural outcome. This is because when EE is put in competition with familiar configuration a dissociation of nearness and shape attributes can occur (e.g., in studies with shaped holes; e.g., Nelson et al., 2014; Peterson, 2003).

In the first experiment, the task instruction was changed to “which side is shaped.” As in Ghose and Palmer (2010), participants viewed stimuli in which EE was on the same side of the shared border as familiarity (congruent stimuli) or on the opposite side of this border (incongruent stimuli). The EE side was crossed with two types of configuration: either an intact familiar configuration or a novel configuration created by spatially rearranging the parts of the familiar configuration. Display orientation was also manipulated: the stimuli were shown both in upright and inverted orientations; upright displays depict the typical orientation of the intact familiar configuration. Four different versions derived from the familiar configurations were used as it has been demonstrated that upright/inverted and intact/part-rearranged configurations have different effects on figure perception (e.g., Barense et al., 2012; Gibson & Peterson, 1994; Peterson et al., 1991; Peterson et al., 1998; Peterson et al., 2000).

For the task with the judgment of nearness, Ghose and Palmer’s (2010) participants had reported the figure on the EE side of the border on 98% of trials in congruent displays and on 92% of trials in incongruent displays; the difference of 6 percentage points was statistically significant (*p* < .001). In Experiment 1, observers were instructed to report which was the shaped side of a border rather than which was the nearer side. EE reports were somewhat lower than those observed by Ghose and Palmer (congruent: 98% vs. incongruent: 95%). The familiarity of the potential shapes mattered: Observers reported that the EE side was the shaped side on 95% (congruent) and 86% (incongruent) of trials with upright displays; smaller congruent/incongruent differences were observed for all other displays. In addition, the difference between EE-side reports on congruent and incongruent trials was larger for the shape task (10 percentage points) than had been shown for the relative near side task by Ghose and Palmer (6 percentage points). Statistical comparisons between the shaped side task set used here and the near side task set used by Ghose and Palmer were not possible, however, because we used a different and larger set of stimuli than Ghose and Palmer, so strong conclusions regarding task set were not possible. Also, note that despite these task-dependent effects, participants primarily reported the EE side as shaped.

In Experiment 2, in addition to asking participants to report which side of the border appeared to be shaped, we added instructions stating that familiar objects might be present.

The EE prior still determined where the figure was perceived on more than 75% of incongruent trials, confirming its dominance as a figural prior. However, the figure was perceived on the EE side of the border on statistically fewer trials in Experiment 2 than in Experiment 1, indicating that participants increased the weight on familiarity in response to the instructions alerting them to the possibility that familiar objects might be present. Notably, however, even PR novel configurations were perceived as the figure more often in Experiment 2 than in Experiment 1. This result was important for two reasons. First, because PR configurations depict novel objects rather than familiar objects (Barense et al., 2012; Flowers et al., 2020; Gibson & Peterson, 1994; Peterson et al., 1991; Peterson et al., 1998; Peterson et al., 2000), this result demonstrates that the Experiment 2 instructions did not simply cause participants to look for familiar objects and report them as figures when they were found. Second, this result suggests that in response to the instructions regarding the possible presence of familiar objects, participants upweighted familiar part responses as well as familiar configuration responses. This finding is new and unexpected. Previous evidence that improperly configured parts activate representations of familiar configurations was observed previously in two conditions only: (1) when participants with damage to the PRC of the MTL were tested and (2) when part-rearranged novel displays were found to prime familiar configurations comprising the same parts (Cacciamani et al., 2014). One possibility is that the instructions directing attention to familiarity altered the PRC mediated top-down modulation of lower-level-part responses proposed by Barense et al. (2012); Cacciamani et al., 2017; Peterson et al., 2012, b). Consistent with this possibility, Sakai (2008) showed that the interaction between brain signals from higher-order cognitive areas and lower-order sensory areas can be modulated by task sets.

The instructions regarding the potential presence of a familiar object included the phrase, “Pay attention.” Given that attention has been conceptualized in numerous ways (Hommel et al., 2019), it is to be noted that here “attention” may be operating to prioritize some stimulus attributes before the outcome of perceptual organization is determined, a mechanism that can be described as “priming.” It has been hypothesized that attention can operate by prioritizing certain stimulus attributes (Gottlieb, 2012; Peterson et al., 2017; Shomstein, 2012; Shomstein & Yantis, 2004). Our data support the hypothesis that the instructions to pay attention to the potential presence of familiar shapes upweighted the influence of familiar parts as well as familiar configurations. Taken as a whole, the three experiments presented here provide the first evidence that the weights on figural priors regarding relative distance versus shape can be influenced by a small change in task set and instructions.

Experiment 3 used a within-subjects design in which participants performed both the “shaped-side” and the “nearer-side” task in two counterbalanced blocks within both halves of the experiment. The halves were defined by the presence (Experiment 3b) or absence (Experiment 3a) of instructions alerting participants to the possibility that familiar objects were present. Thus, Experiment 3a was similar to Experiment 1 in that it was marked by the absence of familiarity instructions, and Experiment 3b was similar to Experiment 2 where the instructions emphasized the possible presence of familiar objects.

In an overall ANOVA on Experiment 3, an effect of task set was evident in a significant two-way interaction between task set and congruency: The EE responses showed a larger difference between the congruent and incongruent versions in the shaped-side task (93% vs. 83%) than in the nearer-side task (94% vs. 87%). The mean difference between EE responses for the shaped versus nearer task was significant for the incongruent condition (83% vs. 87%), but not for the congruent condition. Thus, in Experiment 3, an effect of task set was evident when the two figural priors compete in the incongruent condition, but not when competition is absent in the congruent condition. In the latter condition, reports of the EE side as the figure may have been at functional ceiling, precluding the possibility of measuring task set effects in that condition. The results of Experiment 3 further support the claim regarding the importance of task set made in the discussion of Experiment 1, this time under conditions where task set was manipulated within subjects.

In Experiment 3b, the presence of familiarity was emphasized at the outset as in Experiment 2. In both experiments, these instructions brought out an influence of familiar parts on figure assignment in incongruent PR displays as well as in incongruent intact familiar configuration displays. In Experiment 3b, these instructions regarding the potential of perceiving familiar objects also brought out a congruency effect in inverted displays as well as upright displays. Neuroscience studies have shown that part responses are less orientation specific than whole responses (Baker et al., 2002). Effects that were not observed in Experiment 2 may have been observed in Experiment 3b because of the repetition of displays in the within-subjects design where Experiment 3b always followed Experiment 3a. The presence versus absence of instructions emphasizing the potential of perceiving familiar objects could not be counterbalanced across observers.

Might the instructions in Experiments 2 and 3b simply lead participants to change their strategy such that they no longer reported their first figure–ground organization and reported a reversed figure–ground organization when they did not see a familiar object as the figure? This interpretation is infeasible for a number of reasons. First, the participants were clearly instructed to report their first impression. None of the participants was excluded for indicating on postexperiment questionnaire that they had not done so. Second, the PR configurations appear novel to observers, and hence the instruction effects operated before figure assignment occurred.

These experiments confirm that EE is a dominant figural prior regardless of whether instructions emphasize nearness or shape. At the same time, they show that instructions can change the weighting of figural priors operating outside of awareness. In future research, it will be interesting to investigate the neural mechanisms underlying the differential effects of task set and instructions regarding the possibility of perceiving a familiar object. Although both increase the contribution of familiarity to figure assignment, one does so by highlighting the shape attribute of figures with no reference to familiarity, whereas the other does so by conveying the potential presence of familiar objects.

## Footnotes

1 Such criteria are regularly used for removing data from participants who are outliers.
